# Retrofittable Flexible Fabric Liners with Surface-Functionalized Electroless Nickel Coatings for Midstream Transportation of Bitumen

**DOI:** 10.1021/acs.energyfuels.5c01358

**Published:** 2025-06-06

**Authors:** Joseph K. Cantrell, Lacey D. Douglas, Victor H. Balcorta, Jason A. Weeden, Shruti Hariyani, Rachel H. Lee, James A. Eaves, Kaylyn Stewart, Noah Pieniazek, Matt Pharr, Andrew A. Ezazi, Sarbajit Banerjee

**Affiliations:** † Department of Chemistry, 14736 Texas A&M University, College Station, Texas 77842-3012, United States; ‡ Department of Materials Science and Engineering, Texas A&M University, College Station, Texas 77843-3003, United States; § Quiddity Products, 34011 Sunset Lane, Brookshire, Texas 77423-8517, United States

## Abstract

Increasing global energy demands have heightened reliance on unconventional fossil fuels such as heavy oil and bitumen. However, the rheological properties of these liquids make them challenging to handle and transport. Midstream transportation methods for heavy oil and bitumen encompassing rail cars, trucks, tankers, and pipelines, require extensive dilution with lighter oils and thermal jacketing to facilitate fluid flow. Designing surfaces that readily glide heavy oil would allow for much more efficient midstream transportation by reducing reliance on diluents, decreasing product loss, substantially diminishing maintenance from surface fouling, and alleviating the need for expensive thermal infrastructure. Surfaces that form plastronic air pockets based on reentrant curvature and appropriate surface functionalization show promise for heavy oil drag reduction. Here, we present the design of a superhydrophobic and superoleophobic coating integrated directly onto cotton fabric substrates that can be used to retrofit current midstream infrastructure to glide bitumen. A multiscale hierarchically textured surface is achieved by embedding polytetrafluoroethylene (PTFE) nanobeads within an electroless nickel alloy coating whose surface energy is further reduced by surface functionalization with 1*H*,1*H*,2*H*,2*H*-perfluorooctanephoshonic acid (PFOPA). The wettability of the coated fabric has been examined as a function of coating thickness and surface functionalization for water, light oil, and bitumen. Robust superhydrophobic and superoleophobic behavior is observed with contact angles approaching 162 ± 5° for water and 153 ± 5° for heavy oil. Furthermore, beyond droplet behavior on surfaces, these substrates readily glide bitumen upon immersion. The coated fabric is also thermally and mechanically robust, exhibiting an increase in break force by approximately 20 kN upon addition of the coating and thermal stability up to 250 °C. The surface-functionalized ultrathin electroless nickel coatings integrated onto fabric can be fashioned into different geometric configurations and used as liners in midstream transportation vessels for facile handling and transport of heavy oil. The results demonstrate a promising approach to alleviating some of the most challenging aspects of heavy oil use in the fuel mixes of modern economies.

## Graphical abstract

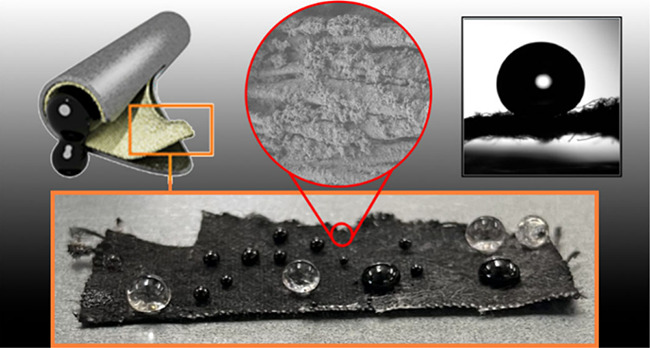

## 1 Introduction

Increasing global demand for energy has resulted in increasing worldwide reliance on unconventional hydrocarbons such as heavy crude oil and bitumen as major contributors to the fuel mix of modern economies.^1–4^ The extraction, midstream transportation, and processing of hydrocarbons from unconventional deposits is challenging because of their complex rheological properties and often high sulfur content.^3,5–7^ Heavy crude is typically either diluted with light hydrocarbons and/or heated to facilitate flow; the latter necessitates an extensive thermal jacketing infrastructure for conduits such as pipelines, rail cars, and tankers.^3,8–12^ As a result of geopolitical happenstance, the geological deposits for bitumen are located at a significant distance (e.g., in the Athabasca region of Canada or along the Orinoco River in Venezuela) from the refineries and processing facilities that are best equipped (such as in the Greater Houston area) to convert such hydrocarbons into a diverse slate of products.^3,13^ Substantial modification of rheological properties is required to facilitate midstream transportation of heavy oil, which is formally classified as having a viscosity in excess of 300,000 cP, API gravity <22.3°, and specific gravity in excess of 920 kg/m^3^.^3^ The addition of diluents, typically light condensates, squanders approximately 30% of pipeline capacity, incurs substantial cost, and entails additional energy-intensive separation steps at refineries to recover heavy oil for further processing.^3^

Other challenges attributable to the high viscosity of viscous oil include challenges in maintenance and cleaning of midstream infrastructure, as well as the loss of substantial product volume (estimated to be as much as 10% in some cases) as a result of surface residues and fouling. In addition, much of the midstream infrastructure is constructed from base metals such as low alloy steels, which are prone to corrosion as a result of the high concentration of sulfur-containing compounds, abrasive particles, and highly corrosive species present in viscous crude oil.^3^ Corrosion-related failures can have a devastating impact on human health and vulnerable ecosystems.^4,5,14,15^ As such, there is growing interest in the design of multifunctional coatings that mitigate surface fouling and enable the facile transfer of viscous liquids.^10–12^ Given the complex form factors and dimensions of midstream transportation vessels, spanning the range from tanks in shipping barges to modular bitutainers, and in light of the difficulties in on-field/on-board application of complex coatings, the design of flexible fabric liners represents an attractive solution for retrofitting current midstream infrastructure.^16^ Such liners could be applied to a variety of containers, including bitumen tank trailers for trucks and cargo bitutainers compatible with transportation by rail and ocean-going vessels from barges to supertankers. Bitutainers in particular represent a significant modular transportation unit whose modification would ease heavy oil transportation. Such containers are a means of bulk transportation of bitumen and asphalt on heated cargo ships such as the barge designs destined for small ports to >250,000 dead weight tonnage used for transcontinental transport and to larger ports.^17,18^ The heating expenses incurred in the transportation of bitumen on these barges amounts to millions of dollars per day. Alleviating these costs would represent a substantial benefit in terms of cost and energy efficiency.^3,17,18^ Here, we describe the design of oleophobic coatings integrated onto cotton fabric surfaces that can be used in retrofitting applications across different forms of midstream storage and transportation vessels.

The design and manufacturing of surfaces that glide and are not wetted by oil droplets represents a considerably greater challenge as compared to the design of surfaces repellant to water as a result of the much lower cohesive forces (primarily, dispersive interactions) and thus much lower surface tension of the former liquid.^19^ Three key aspects to controlling the behavior of liquid droplets and flowstreams on engineered surfaces based on modulation of fluid/solid interfacial interactions include: texturation spanning a hierarchy of length scales; 3D topgraphical features that define reentrant curvature; and modulation of surface energy as governed by pendant chemical moieties that encounter an impinging liquid.^3^ Under appropriate conditions, a fluid droplet can be suspended in the metastable Cassie—Baxter regime, wherein it is sited atop a topographically textured surface and over trapped pockets of air known as plastrons.^20,21^ We have previously demonstrated both superhydrophobic and/or superoleophobic behavior utilizing ZnO tetrapods on metal meshes as textural elements, and based on using the low-temperature sintering of TiO_2_ nanoparticles arrayed onto solid steel coupons by colloidal crystal templating.^11,12,16^ However, the integration of these coatings with current transportation vessels is constrained by challenges in thermal stability, durability, mechanical resilience, and adhesion of the coatings, compounded by difficulties with field application. These limitations have restricted the viability of such coatings in retrofitting existing midstream transportation infrastructure. Here, we demonstrate integration of a superhydrophobic and superoleophobic electroless nickel composite coating on a cotton fabric surface that allows for facile integration within existing transportation vessels and can be readily fitted to adopt various form factors.

Electroless plating has found extensive industrial applications on planar metal surfaces since the first report by Brenner and Riddell.^22^ Electroless nickel coatings exhibit a combination of excellent corrosion inhibition, as well as wear and abrasion resistance, homogeneous thickness across extended length scales, excellent adhesion to a variety of substrates, and applicability across a diverse range of form factors.^23^ Electroless nickel formulations typically contain a source of nickel ions, a reducing agent, complexing agents, and stabilizers. The autocatalytic reaction for electroless nickel deposition can be written as

1 $$5H_{2}{{\mathrm{PO}}_{2}}^{-}\left(\mathrm{aq}\right)+{\mathrm{Ni}}^{2+}\left(\mathrm{aq}\right)\to 3H_{2}{{\mathrm{PO}}_{3}}^{-}\left(\mathrm{aq}\right)+\mathrm{Ni}\left(s\right)+2P\left(s\right)+H_{2}O\left(l\right)+H_{2}\left(g\right)$$

Electroless nickel coatings are amenable to alloying, as well as the inclusion of bulk and surface precipitates. Nickel alloying is accomplished by the incorporation of phosphorus or boron from the reducing agent; the volume of incorporated phosphorus influences the grain dimensions and extent of crystallinity of the electroless nickel coatings. The incorporation of nanoparticles facilitates the formation of composite coatings with tailorable properties.^24,25^ For example, the inclusion of hard particles such as diamond or soft particles such as fluorinated compounds can alter the lubricity of the coating. Guglielmi’s theory describes the mechanism by which particles can be embedded during electroless deposition. Adsorption occurs in two sequential steps, which establishes a relationship between the ultimate particle concentration embedded within the coating and particle concentration in the precursor bath dispersion.^26^ In this construct, the dispersed inert filler particles in the plating solution are adsorbed to the surface by fast, reversible physical interactions.^26,27^ Next, the redox plating reaction deposits product around the inert particles, which thereby affixes the particles to the incipient plated layer. The rate of the second process is dependent on the plating reaction rate, but the number of particles available to be incorporated within the composite coating is held approximately constant by the requirement that the particles must physically adsorb first.^26,27^

In this article, we use electroless deposition to coat a cotton fabric with a nickel phosphorus alloy incorporating polytetrafluoroethylene (PTFE) beads; next, the coating is functionalized with 1*H*,1*H*,2*H*,2*H*-perfluorooctane phosphonic acid (PFOPA). The composite textured and low-surface-energy coating enables the rapid removal of heavy oil and water and retains superhydrophobic and superoleophobic properties after mechanical deformation. The ability to fabricate thermally robust and mechanically resilient large-area fabric substrates to exhibit robust omniphobicity provides an innovative retrofittable solution to challenges with viscous oil handling in the midstream sector.

## 2 Materials and Methods

### 2.1 Coating Fabrication

Parchment-colored 100% utility cotton (JOANN Fabrics & Crafts) was chosen as the substrate for these coatings. The initial fabric activation step was performed by adapting a procedure described in the literature.^28^ A cotton fabric substrate was submerged in a 1 wt % ethanol (96%, Fisher Chemical) solution of 3-aminopropyltrimethoxysilane (APTMS, 97%, Sigma) at room temperature for 24 h. Next, the substrate was removed from the solution, annealed at 70 °C for 1 h in a muffle furnace (Thermo Scientific Thermolyne FD1540M), and rinsed with deionized water (Thermo Scientific Barnstead GenPure filtration system, ρ = 18.2 MΩ·cm^–1^) using a spray bottle. Subsequently, the sample was immersed in a 0.05 wt % PdCl_2_ aqueous solution with 0.2 M HCl at room temperature for 10 min, followed by rinsing with deionized water, ca. 2—5 mL per square inch. Next, the activated substrate was submerged in Caswell electroless nickel plating (EN) solutions at 85—90 °C (Caswell, Inc., Lyons, NY, USA) for various durations ranging from 1—60 min. The solution was replenished every 5—10 min; PTFE beads were added as needed to obtain thicker coatings. Upon removal from the EN-PTFE bath the coated fabric was rinsed with DI water with a spray bottle until the runoff was clear. Next, the coating was immersed in a 27 mM solution of 1*H*,1*H*,2*H*,2*H*-perfluorooctane phosphonic acid (PFOPA, 95%, Sigma) in ethanol for 24 h. The final coating is schematically illustrated in Figure 1.

**Figure 1:**
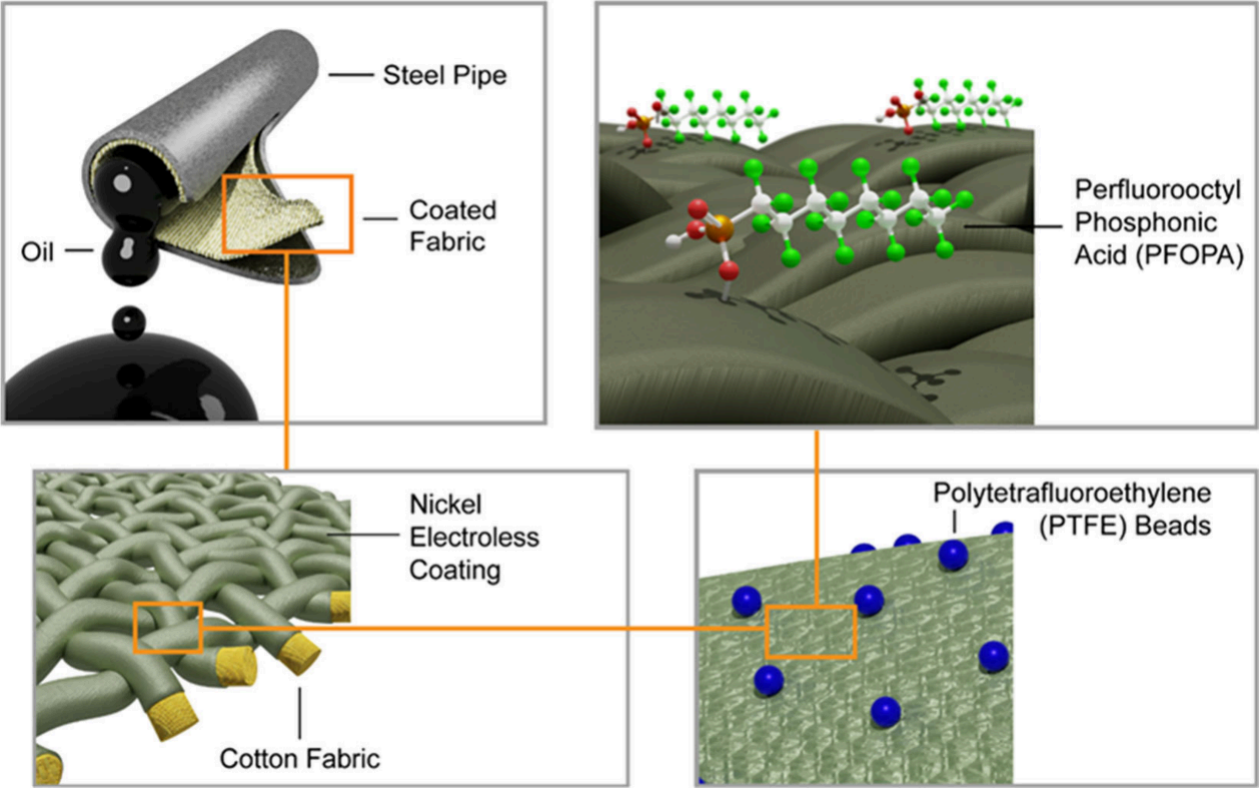
Workflow for integrating an electroless nickel composite coating onto cotton fabric. Electroless nickel electrodeposition embedding PTFE beads yields a conformal surface coating on fibers of a woven cotton fabric. The nickel composite coating is further functionalized with PFOPA to imbue oleophobic properties.

### 2.2 Hydrocarbons

Substrates were tested using water and two types of oil. Light sweet crude oil with a specific gravity of 0.8050 g/mL was obtained from the Texas Permian Basin.^29^ Heavy polymer-modified bituminous oil was obtained from Puma Energy’s Australian Altona terminal. The viscosity of the heavy oil sample was measured as ca. 140 cP at 150 °C using a rotational rheometer with 40 mm parallel Peltier plate (Discovery Hybrid DHR-2 rheometer, TA Instruments).

### 2.3 Coating Characterization

Wettability of the coated substrates was evaluated by measuring contact angles using a goniometer (Attension Theta Lite). The values reported are an average of a minimum of measurements taken across three distinct areas across the substrate. Approximately 20 μL of deionized water and light sweet crude oil were dispensed onto substrates manually prior to recording a digital image. Heavy oil (Puma Energy) droplet contact angles, were measured by manually placing oil droplets of ca. 20 μL onto the substrate and analyzing the droplet using the Attension Theta Lite software. Heavy oil contact angles were recorded at temperatures of 175 °C, by heating the heavy oil to the target temperature and placing the substrate on a hot plate (VWR VMSC4) set to the same temperature. All other measurements were acquired at room temperature unless otherwise denoted. Dynamic contact angles were measured with the same goniometer with a changing drop size of 0—20 μL at a rate-of-change 0.5 μL/s (40 s advancing measurement and 40 s receding measurement).

Roll-off angles were obtained with 20 μL droplets based on two methods. In the first approach, the “tilting plate method”, droplets were dispensed onto a flat fabric surface and the surface angle changed at approximately 1°/s until translational motion of the droplet is initiated with reference to a marked location on the fabric to ensure objectivity.^30^ Multiple droplets were tested at different fabric surfaces. In the second approach, labeled as “centimeter drop”, droplets were also dispensed ca. 1 cm above a fabric substrate angled at 15° and the angle decreased at approximately 1° per droplet until the drop did not roll off on contact.

The surface morphology of the coated substrates was examined using field-emission scanning electron microscopy (FE-SEM) (JEOL JSM-7500F) with an emission current of 10 μA, probe current of 8 μA, accelerating voltage of 5 kV, and 15 mm working distance to the pole piece. Bare fabric substrates were mounted on Cu tape and coated with 5 nm of Pt using a 208HR High-Resolution Sputter Coater. Energy dispersive X-ray spectroscopy (EDS) measurements were recorded using the Oxford system with an accelerating voltage of 5 kV, emission current of 20 μA, probe current of 12 μA, and a working distance of 8 mm.

To determine the thickness of the coatings, cross-sectional samples were prepared for SEM by a previously established procedure.^31^ Fabric pieces were embedded in Epoxicure 2 resin and hardener (4:1 ratio) and left at room temperature to harden for 24 h. A cut through the embedded fabric was made with a Buehler IsoMet diamond precision saw and the cut surface was then ground with a Buehler EcoMet 30 grinding and polishing wheel using 1200 grit P600 silicon carbide sandpaper followed by 4000 grit P1200 silicon carbide sandpaper. Polishing was then done with an Electron Microscopy Sciences 1 μm diamond polishing paste and 200 mm Struers MD-Floc polishing pads. The polishing paste was diluted with Falcon Tool Company water-based polishing lubricant and diamond thinner. The samples were sputter coated with 5 nm of Pt and examined under FE-SEM and EDS as described above. A minimum of three coating replicates were examined at three different locations to determine the thickness of the deposited nickel coating, and errors were determined by recording the standard deviations of thicknesses of the thread and fractionally propagating that to the coating thickness over these different samples and locations.

X-ray photoelectron spectroscopy (XPS) was recorded with an Omicron DAR 400 XPS/UPS system with a 128 microchannel Argus detector. A 1253.6 eV Mg X-ray source at 15 kV and 20 mA emission current were utilized with a CN10 electron source to minimize charging. Spectra were calibrated to a carbon 1 s feature from adventitious carbon at 248.8 eV. Fabric samples were kept in an Across International model AT19 vacuum oven at 100 °C for 2 days prior to measurement.

A Bruker Vertex-70 with PIKE MIRacle single-reflection horizontal attenuated total reflectance (ATR) accessory was used to acquire Fourier-transform infrared (FTIR) spectroscopy data. Sample substrates were pinched underneath the sample head above a diamond ATR crystal. Thermogravimetric Analysis (TGA) data were collected using a TA Instruments TGA 5500 at a ramp rate of 20 °C/min up to 900 °C. In a typical experiment, a 3–7 mg sample was placed in a platinum pan under an inert atmosphere.

Tensile testing was performed following ASTM D5035 to evaluate the mechanical properties of textile fabrics after coatings were applied for 1, 10, and 60 min, alongside uncoated control samples.^32^ Testing parameters were selected based on the results of textured fabric tensile studies reported in the literature.^33^ Specimens were precisely cut using a rotary cutter to prevent edge distortion and conditioned at room temperature (22 °C) before testing. A 1 kN Instron 5943 tensile tester with pneumatic side-action grips was used, applying a displacement rate of 100 mm/min. The gauge length between grips was set at 70 mm, and samples were stretched to rupture, recording breaking force (in N) and elongation at maximum force. At least 15 replicates were tested per sample type to ensure statistical robustness. Data were analyzed for statistical significance, with results reported as mean values and standard deviations.

## 3 Results and Discussion

### 3.1 Characterization of Coating Morphology, Surface Functionalization, Thermal Stability, and Mechanical Resilience

Figure 1 schematically illustrates the deposition of an alloyed Ni composite coating onto woven cotton fabric. Initial surface activation is achieved using APTMS and PdCl_2_, followed by electroless deposition of a nickel composite coating embedding PTFE beads for various times (as denoted in sample labels), followed subsequently by surface functionalization with PFOPA. Based on analogous experiments performed on flat low-alloy steel substrates, electroless nickel coatings are observed to provide smooth conformal deposition; the addition of ca. 200 nm PTFE beads yields agglomerated beads dispersed across the surface (Supporting Information Figure S1).

High-resolution scanning electron micrographs in Figure 2 show the intricate woven pattern of the cotton fabric (bare cotton fibers are shown in Figure S2) and attest to the incorporation of electroless nickel and PTFE on each individual thread of the substrate. Contrasting the bare cotton fabric at varying magnifications with the coated substrates, PTFE nanobeads are embedded along each strand. Some agglomeration of the PTFE beads in clusters is observed at the intersections of the woven pattern (Figure 2C). PTFE nanobeads are observed to agglomerate into complex texturizing elements at lower deposition times, as seen in Figure 2A–F. The appearance of the texturation across three different magnifications, with EDS overlays, is exhibited in Figure S3, and the corresponding EDS spectra are shown in Figure S4. After 10 min of electroless deposition, larger PTFE agglomerations are observed, as exhibited in Figure S5. With increased coating thickness, it becomes more difficult to retain the PTFE bead texturization as it is overcoated by the electroless Ni alloy (Figure 2G,H) as expected by Guglielmi’s model discussed above. This “smoothening” effect is especially pronounced for reaction times more than 45 min.^10^ The corresponding EDS maps of the texturizing elements in Figure 2 are shown in Figure S6 alongside their corresponding EDS spectra.

**Figure 2:**
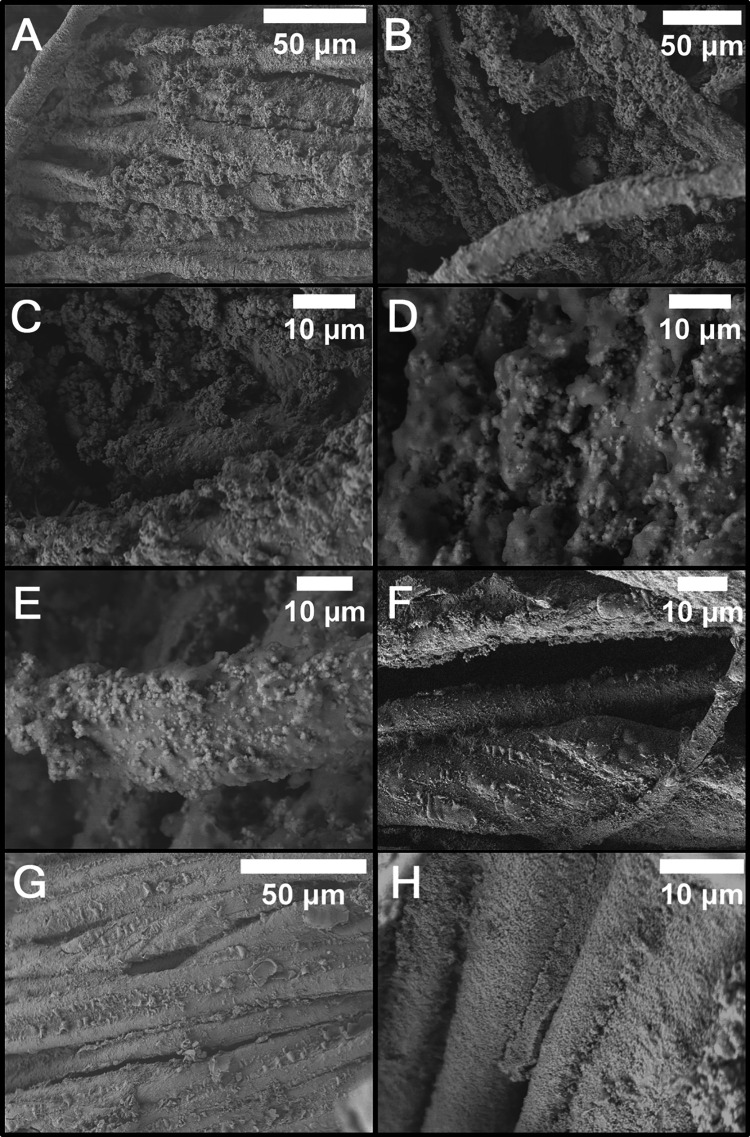
Surface texturation of coated cotton fabrics. Scanning electron micrographs and EDS maps of EN-PTFE coated cotton fabric samples with PFOPA functionalization. (A—F) Coatings with 1 min of EN-PTFE coating and PFOPA functionalization. Panels A—D show how complex PTFE texturation can arise from integration of PTFE beads along fibers. Panels E and F are higher magnifications of the beads on the fibers. (G, H) SEM images of texturizing features after 45 min of EN-PTFE coating and PFOPA functionalization. The corresponding EDS maps are shown in Figure S6.

The evolution of coating thickness as a function of electroless deposition reaction time is plotted in Figure 3A. After an initial rapid deposition of about 2 μm of the composite, the coating thickness increases at a steady rate of 0.2 μm/min. The rapid initial growth in thickness is consistent with the electroless reduction, nucleation, and deposition of nickel alloy particles mediated by surface activation of the cotton fibers by reaction with APTMS and PdCl_2_. Beyond the initial nucleation and deposition step, which proceeds until conformal coverage is achieved across the surface-functionalized cotton fibers, there is a direct correlation between the time the cotton fabric substrates are immersed in the PTFE electroless nickel bath and the coating thickness. During deposition times from 1 to 60 min, the deposition thickness steadily increases from ca. 2 to 14 μm (Figure 3A).^10^ To evaluate the thermal stability of the engineered substrates, thermal gravimetric analysis (TGA) has been performed. As shown in Figure 3B, the derivative curve indicates three separate thermal degradation processes. Control TGA experiments have been performed on untreated cotton fabric, cotton fabric with an electroless nickel coating without PTFE beads, cotton fabric with EN-PTFE (but without PFOPA functionalization), and finally cotton fabric with EN-PTFE coating and PFOPA functionalization. Based on these control experiments, the first process in the temperature range of 20–50 °C corresponds to a small mass loss arising from loss of volatile species from the cotton substrate. The second mass loss regime in the temperature range of 260–360 °C corresponds to the degradation of the cotton substrate. The third mass loss regime in the temperature range of 360–460 °C corresponds to the gradual decomposition of the PTFE beads. Since the predominant degradation mechanisms are incurred beyond 220 °C, the highest handling temperature of bitumen, the functionalized fabrics indeed exhibit the desired thermal robustness needed for midstream applications.

**Figure 3:**
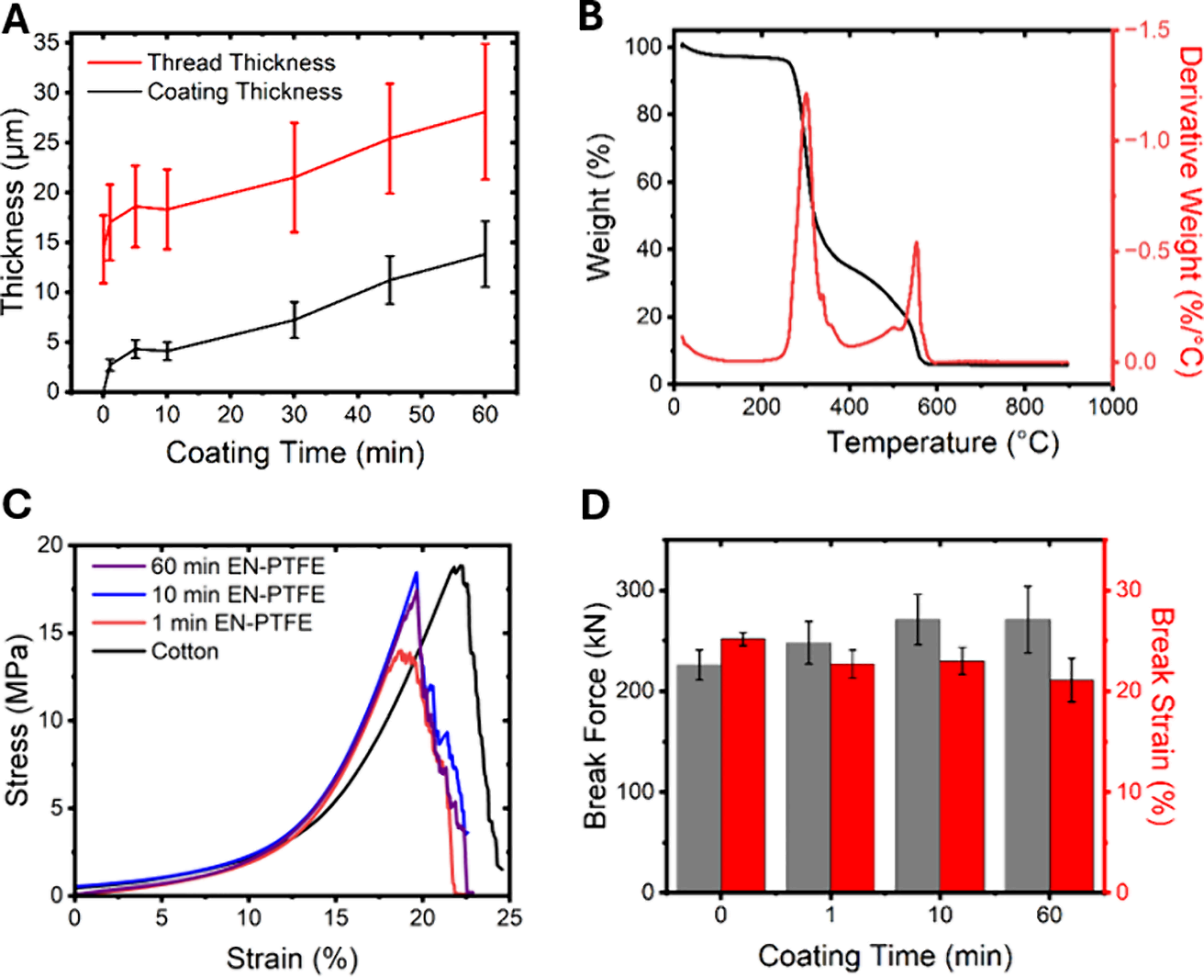
Coating thickness, thermal stability, and mechanical resilience. (A) Evolution of EN-PTFE coating thickness as a function of electroless deposition reaction time. Error bars represent the standard deviation of thickness of the thread and, fractionally, of the coatings, with at least 3 samples observed. (B) Thermogravimetric analysis of EN-PTFE PFOPA functionalized coatings deposited onto cotton fabrics for 10 min. (C) Representative stress—strain curves acquired for bare cotton and EN-PTFE-coated samples at 1, 10, and 60 min of electroless deposition time. (D) Measured break force and break strain of bare cotton and EN-PTFE-coated cotton fabric samples after 1, 10, and 60 min of electroless deposition time. All error bars correspond to standard deviations from measurements.

The mechanical resilience of the coatings have further been evaluated using the tensile testing experiments shown in Figure 3C,D. The results indicate that the EN-PTFE coating increases the breaking force of fabric samples; a progressive increase in breaking force is observed with an increase in coating time. Statistical analyses confirm that all coated samples exhibited significantly higher breaking force as compared to plain cotton (*p* < 0.05), demonstrating improved mechanical strength (Table S1). However, this increase in strength comes at the expense of reduced breaking strain, as coated samples exhibited progressively lower flexibility with longer coating durations. *t* test results in Table S1 confirmed that the reduction in elongation was also statistically significant (*p* < 0.05), which indicates a trade-off between strength and flexibility. These findings suggest that while the coating reinforces the fabric, it also makes it less deformable, a factor that will need to be considered in designing liners for bitumen vessels with different form factors and geometries.

Figure 4 presents spectroscopic characterization of the functionalized fabrics. Figure 4A contrasts the distinctive vibrational modes of the uncoated cotton substrate, cotton substrate after EN-PTFE deposition, and the EN-PTFE coated substrate after functionalization with PFOPA. A FTIR spectrum acquired for PFOPA is also shown for comparison. The appearance of characteristic fluoroalkyl modes observed in the functionalized fabric sample corroborates PFOPA functionalization of the Ni surface. Specifically, bands at 1142, 1184, and 1207 cm^–1^ are assigned to symmetric −CF_2_, asymmetric −CF_3_, and asymmetric −CF_2_ stretches, respectively.^34–36^ The 1232 cm^–1^ band is derived from overlapping symmetrical −CF_2_ and −P=O stretches.^34,35^ A blue shift is observed for the P=O and P–O bands from 952, 935, and 920 cm^–1^ for the free molecule to 995, 977, and 962 cm^–1^ in the surface-bound species, which corroborates the grafting of PFOPA to Ni surfaces through phosphonate head groups.^34,35^ Similar changes in PFOPA vibrational modes have been ascribed to formation of a fluorocarbon helix on the surface.^10,11^ Figure 4B plots the evolution of ATR-IR absorbance with varying EN-PTFE deposition times. Notably, the water IR bands at 1350 and 3700 cm^–1^ are greatly diminished after 2 min of coating and the cotton bands (such as at 1000, 2900, and 3300 cm^–1^) are no longer discernible after 10 min of coating. The latter reflects the complete coverage of the cotton fibers by the composite Ni alloy coating.

**Figure 4:**
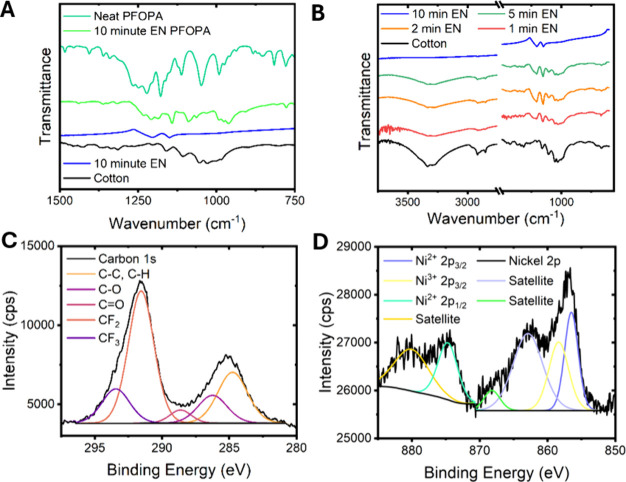
Spectroscopic characterization of surface functionalization. (A) FTIR transmission spectra before and after EN-PTFE deposition and after PFOPA functionalization. A FTIR spectrum acquired for PFOPA is plotted alongside for comparison. (B) Evolution of FTIR transmission spectra as a function of EN-PTFE deposition time. (C) Core-level carbon 1s XPS spectrum obtained for a cotton substrate coated with EN-PTFE for 10 min and subsequently functionalized with PFOPA. (D) Core-level Ni 2p spectrum obtained for the same sample.

Panels C and D of Figure 4 and Figure S7 show core-level XPS spectra of 10 min EN-PTFE coated cotton fabric. The peaks have been assigned based on compiled library values for elements and in comparison to XPS spectra acquired for nickel oxide and phosphide.^37–39^ The surface of the coated fabric shows pendant fluorocarbon moieties derived from the PTFE beads and PFOPA monolayer with a smaller contribution of ether, carbonyl, and alkyl moieties as shown in Figure 4C. As can be seen in Figure 4D, the composite surface comprises predominant Ni^2+^ and Ni^3+^ contributions.^38^ The satellite peaks are either magnetic shake ups from Ni^2+^ or multiplet peaks from Ni^2+^ unpaired electrons, and their lineshapes are characteristic of nickel bound to either oxygen or phosphorus.^40–43^ Figure S7 plots core-level F 1s XPS spectra, which support these ideas by demonstrating the presence of distinctive CF_2_ and CF_3_ features.

### 3.2 Evaluation of Coating Wettability

The wettability of the coated cotton fabrics toward water and bitumen has been evaluated as a function of electroless nickel/PTFE deposition time (which governs the coating thickness and texture) and PFOPA functionalization. The former is key to precise control of the multiscale texturation and reentrant curvature that defines plastronic architectures and mediates the surface topographies that interact with liquids, whereas the latter governs the surface energy at the solid/liquid interface.^44^ Coatings to facilitate bitumen midstream transportation are required to have high contact angles, low roll-off angles, and low contact angle hysteresis.^33^ Three different probe liquids have been used, deionized water, sweet light crude oil, and bitumen. Figure S8 plots the temperature-dependent viscosity of a heavy oil sourced from Puma Energy. Table S2 lists these values for EN-PTFE coatings on cotton fabrics for varying electroless deposition times, whereas Table 1 and Table S3 lists values for EN-PTFE coatings on cotton fabrics after PFOPA functionalization. Ni alloy composite coatings embedding PTFE beads imbue superhydrophobicity even before functionalization with PFOPA (Table S2). Indeed, even the thinnest EN-PTFE coated fabrics show water contact angles >150°. EN-PTFE coatings further demonstrate promising normal oleophobicity toward bitumen at 175 °C, but do flash wet sweet light crude oil, which has a lower viscosity. Notably, the contact angle hysteresis is halved for EN-PTFE coatings as compared to bare cotton and cotton functionalized with PFOPA, which indicates that the combination of 3D texturation and low-surface-energy PTFE inclusions visible in Figure 2 greatly reduce the interactions between water droplets and the substrate.

**Table 1:** Characterization of Wettability^a^

	Water Contact Angle (deg)	Bitumen Contact Angle (deg)	Advancing Water Contact Angle (deg)	Receding Water Contact Angle (deg)	Water Contact Angle Hysteresis (deg)	Tilting Plate Roll-Off Angle (deg)
Bare Cotton	112 ± 3	86 ± 4	143 ± 12	95 ± 16	48 ± 16	>90
Cotton PFOPA	149 ± 2	143 ± 2	157 ± 23	112 ± 14	45 ± 23	>90
1 min EN-PTFE PFOPA	157 ± 2	153 ± 4	153 ± 18	132 ± 17	21 ± 18	29 ± 4
2 min EN-PTFE PFOPA	162 ± 5	150 ± 4	160 ± 13	145 ± 14	15 ± 14	33 ± 5
5 min EN-PTFE PFOPA	158 ± 2	146 ± 4	158 ± 12	148 ± 17	17 ± 17	30 ± 2
10 min EN-PTFE PFOPA	154 ± 9	145 ± 5	154 ± 10	142 ± 7	12 ± 10	30 ± 10
20 min EN-PTFE PFOPA	158 ± 6	153 ± 5	155 ± 12	144 ± 16	16 ± 16	23 ± 3
30 min EN-PTFE PFOPA	160 ± 6	140 ± 4	161 ± 10	149 ± 17	17 ± 17	31 ± 8
45 min EN-PTFE PFOPA	144 ± 4	137 ± 7	150 ± 6	137 ± 10	13 ± 10	35 ± 1
60 min EN-PTFE PFOPA	150 ± 6	136 ± 2	150 ± 4	135 ± 15	15 ± 15	30 ± 5

a Water and heavy oil (synthetic bitumen, Puma Energy) static contact angles measured for different thicknesses of EN-PTFE films with surface functionalization with PFOPA. Dynamic water contact angles (advancing, receding, hysteresis) are also shown. The rightmost column lists water roll-off angles. Deposition times from 1—60 min noted in the first column correspond to EN-PTFE thin film thicknesses from ca. 2 to 14 μm. Values reported are the mean of at least 3 measurements, and error values represent standard deviation. Dynamic and hysteresis values are advancing angles minus receding angles, and thus errors reflect standard deviations in measurement of initial angles. Permian Basin light sweet oil contact angles, dynamic contact angles, and centimeter drop roll-off angles are listed in Table S3. Values measured prior to PFOPA surface functionalization are listed in Table S2.

Next, turning to Table 1 and Figure 5, PFOPA functionalization halves the water contact angle hysteresis again, which reflects a further reduction in surface energy at the liquid/solid interface that decreases interactions between fluid droplets and the coated cotton fabric substrates. Table 1 illustrates that EN-PTFE coatings with PFOPA functionalization glide water droplets at roll off angles between 35° and 50° for coating less than 20 min. As the coating exceeds thicknesses of 5 μm, water droplets no longer roll off quite so easily because of the loss of PTFE-nanobead-derived texturization. Notably, with diminution of geometric texturation as a result of Ni overdeposition around PTFE beads and between individual threads of the woven fibers, the contact angles of water and bitumen are reduced by ca. 10—20°. As such, coating thicknesses of <10 μm corresponding to deposition times <45 min yield optimal EN-PTFE texturation.

**Figure 5:**
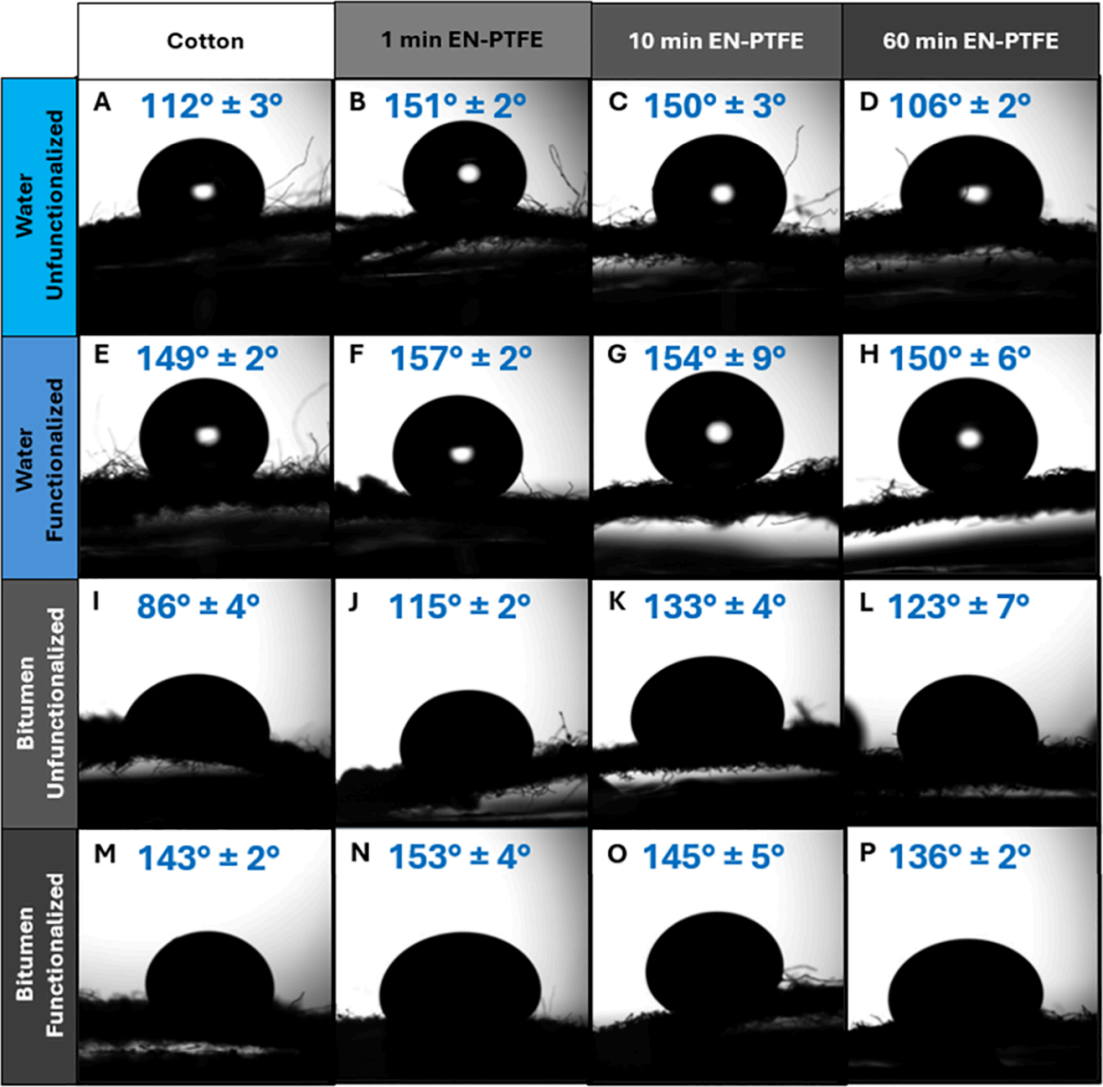
Wettability as evaluated by contact angle measurements. Digital photographs of water and bitumen droplets on a cotton fabric substrate under blacklight with and without nickel coating and PFOPA functionalization. (A—H) Water contact angles and (I—P) bitumen contact angles. Panels A—D and I—L show unfunctionalized coatings, whereas panels E—H and M—P show PFOPA-functionalized coatings. The columns from left to right show the bare cotton fabric substate, EN-PTFE coatings deposited for 1 min, EN-PTFE coatings deposited for 10 min, and EN-PTFE coatings deposited for 60 min.

Examining the wettability metrics listed in Table 1, superhydrophobicity and superoleophobicity is achieved even for the thinnest 1 min EN-PTFE electroless deposition time, which corresponds to a thickness of 2.7 ± 0.6 μm upon surface functionalization with PFOPA at a concentration of 27 mM in ethanol. Figure 5 depicts bitumen and deionized water droplets placed on top of coated cotton substrates. The untreated fabric is hydrophobic and oleophilic to bitumen. The EN-PTFE coating or direct PFOPA functionalization of the cotton substrate achieves superhydrophobicity and bitumen oleophobicity; however, both coating with EN-PTFE and subsequent functionalization with PFOPA are required to achieve bitumen superoleophobocity and light crude oil oleophobicity (Figure 5N). In general, a decrease in oleophobicity is observed beyond coating times of 45 min with increasing thickness of the coatings, as Ni deposits into intervening crevices and smooths the asperities between the individual threads of the woven fabric substrate, thereby bringing about an overall reduction in the different scales of texture (as evidenced in Figure 2N–O). An optimal combination of water and oil repellence is observed for EN-PTFE samples less than 5 μm (corresponding to deposition times <5 min), which show contact angles of 162 ± 5° for water, 153 ± 4°for heavy oil at 175 °C, and 124 ± 2° for light sweet crude oil.

Perfluorooctanoic acid (PFOA) and an order of magnitude smaller concentration (2.7 mM) of PFOPA were also tested as functionalizing procedures of the EN-PTFE coatings (Table S4). These coatings also achieved superhydrophobicity and superoleophobicity in very similar fashion to the 27 mM PFOPA coatings shown in Table 1. However, the 2.7 mM PFOPA functionalized samples suffered a loss of 10° in heavy contact angles as compared to the best performing 27 mM PFOPA functionalized samples, which is likely indicative of submonolayer PFOPA coverage of surfaces contacted by oil droplets.

Dynamic contact angle measurements provide a glimpse of the evolution of substrate wettability upon liquid contact. Videos S1, S2, and S3 show dispensed water droplets during advancing and receding contact angle measurements for bare cotton, cotton fabrics directly functionalized with PFOPA, and EN-PTFE fabrics after further functionalization with PFOPA. When measuring dynamic contact angles of the substrates, wetting over time is observed for all fabric samples not coated with EN-PTFE. Bare cotton wets in less than a minute despite initially exhibiting a nonzero contact angle; direct PFOPA functionalization of the substrate delays water wetting, which is nevertheless observed after ca. 5 min of placement of a water droplet. This wetting is particularly notable in measurements of receding contact angles, as shown in Figure 6. In stark contrast, a PFOPA-functionalized EN-PTFE coating is so water repellent that the water droplet is pulled completely off the substrate by the pipet.

**Figure 6:**
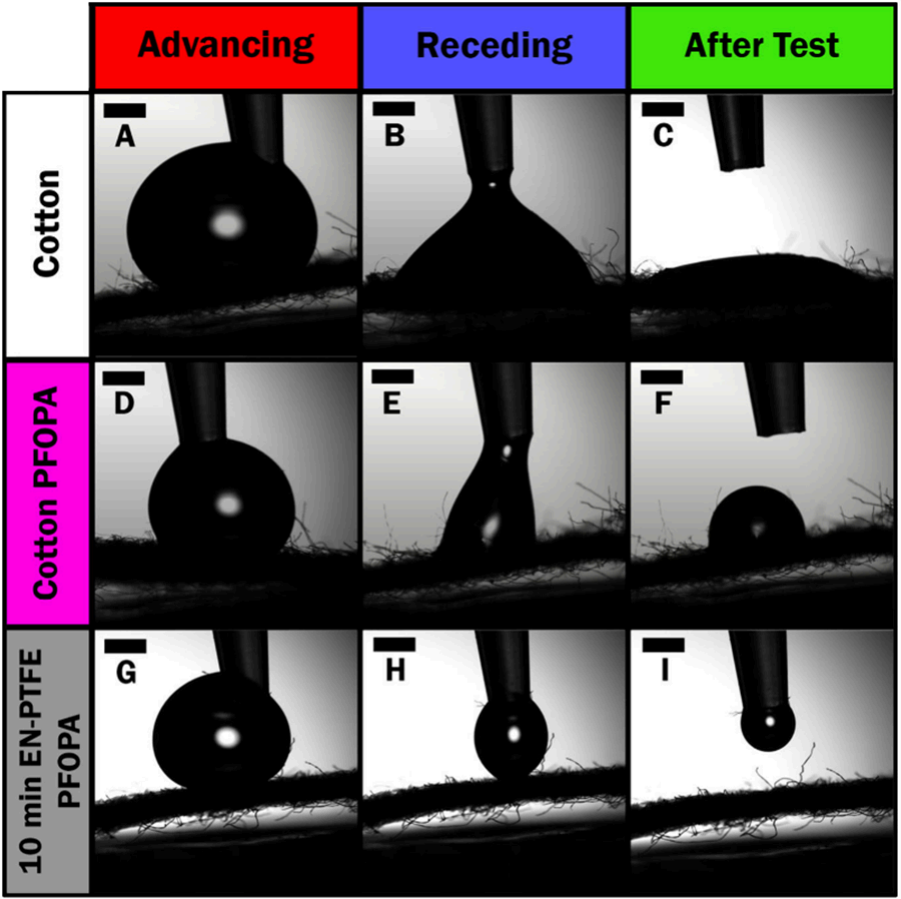
Wettability as evaluated by dynamic contact angle measurements. Digital images of water droplets dispensed and retracted on uncoated and coated cotton fabric substrates under blacklight. (A—C) Dispensation of a water droplet onto a bare cotton fabric and ensuing wetting of the substrate. (D—F) Dispensation of a water droplet onto a cotton fabric directly functionalized with PFOPA but without an EN-PTFE coating, which results in delayed but eventual wetting. (G—I) Dispensation of a water droplet onto an EN-PTFE-coated fabric with an electroless deposition time of 10 min subsequently functionalized with PFOPA. The substrate strongly repels water droplets, which are retracted into the dispenser. In each case, the left column shows advancing contact angles, the middle column shows receding contact angles, and the right column shows the wetting behavior immediately following the receding measurement. Scale bars represent 1 mm. Full videos are shown in Supporting Information Videos S1, S2, and S3.

We next go beyond individual droplets and examine the wettability of coated substrates upon immersion in water and bitumen, as relevant to the midstream transportation of bitumen. The EN-PTFE-coated fabric with PFOPA functionalization is subjected to several tests involving immersion within water or bitumen, or where these liquids were flowed across the substrates (Videos S4, S5, S6, S7, S8, and S9). Stills abstracted from the videos contrasting the wettability of bare cotton and the EN-PTFE-coated fabric with PFOPA functionalization are shown in Figure 7. In all cases, water and bitumen were removed from the latter substrate within 30 s with remnants being pinned that still do not wet the substrate.

**Figure 7:**
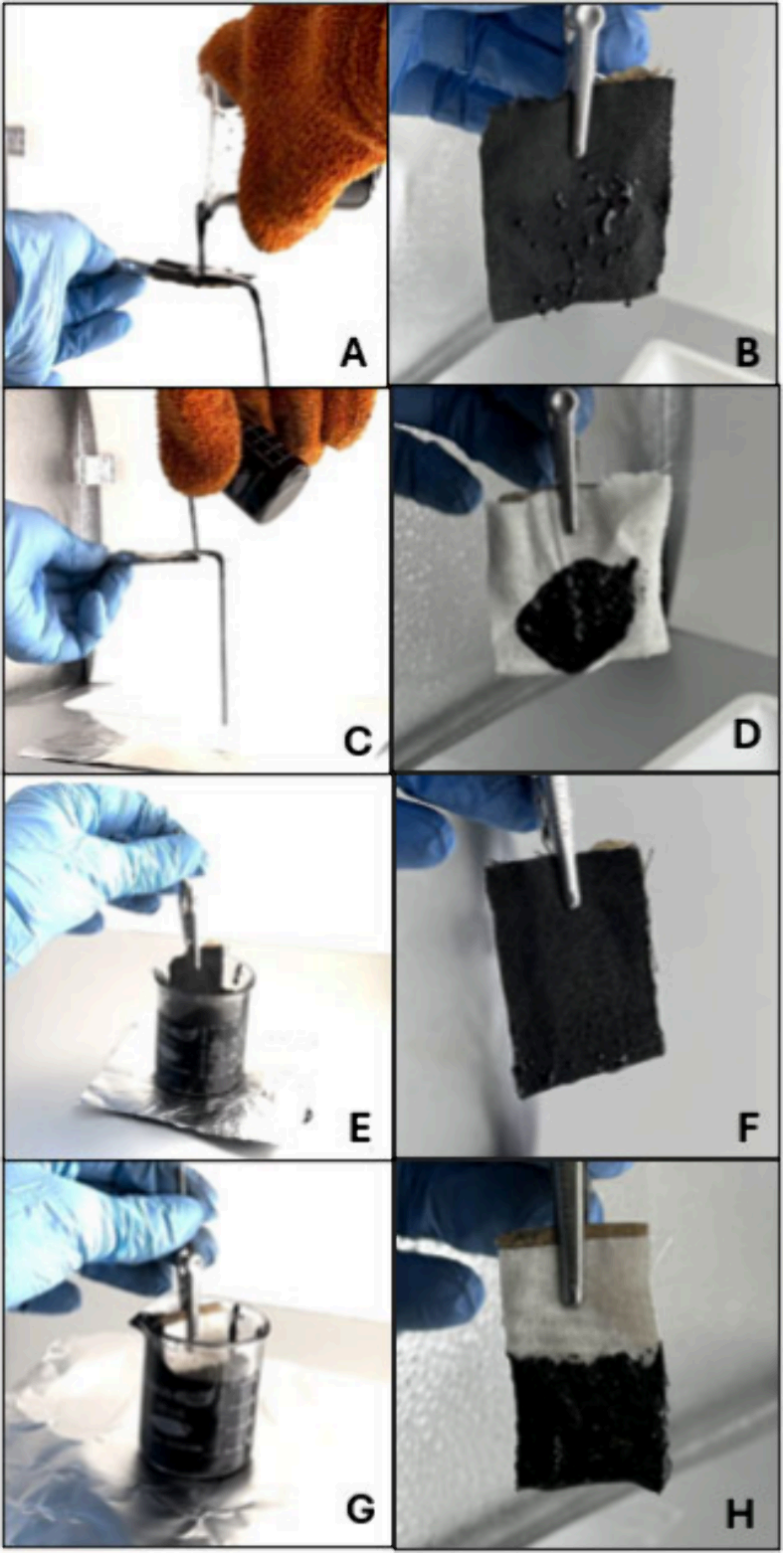
Examination of bulk bitumen wettability. Still digital images contrasting the wettability of EN-PTFE-coated coated fabric subsequently functionalized with PFOPA (A, B, E, and F) and bare cotton (C, D, G, and H) toward bitumen. (A, B) Heavy crude oil is poured onto an EN-PTFE-coated fabric subsequently functionalized with PFOPA; (C, D) heavy crude oil is poured onto a bare cotton fabric. (E, F) EN-PTFE-coated fabric subsequently functionalized with PFOPA is immersed in heavy crude oil; (G, H) bare cotton fabric is immersed in heavy crude oil. Stills are taken from Videos S4, S5, S6, and S7.

A cotton substrate with an EN-PTFE coating and PFOPA functionalization was fashioned into an open-faced cubic receptacle and filled with deionized water and heavy oil as a scale model for a bitutainer. The wettability toward water at 25 °C and heavy oil at 150 °C was examined for these scale models. In both instances, the liquids were rapidly removed from the receptacle and the coated fabric was readily recovered in near pristine condition. The coated receptacle was further filled with water and was allowed to stand for 1 h. No outflows or leaks of water were observed, and the receptacle fully preserved its ability to repel water. These results indicate the robustness of the plastrons that are stabilized from the multiscale texture, reentrant curvature, and reduced surface energy resulting from embedding PTFE beads and PFOPA functionalization. Indeed, the hydrophobic and oleophobic characteristics of the coatings were maintained for over 6 months under ambient conditions. These results further indicate that ultrathin EN-PTFE coatings remain flexible and readily glide heavy oil even after being shaped into different configurations, which attests to their suitability for application as liners for retrofitting midstream transportation vessels.

To validate the ability of these coatings to retain heavy oil without leaks over an extended period of time, a 45-day study was conducted with bitumen at 175 °C. Three EN-PTFE-PFOPA-coated cotton fabrics with dimensions of 2 in. × 4 3/8 in. were folded to create a boat shape as shown in Figure S9. The boats were suspended in a 600 mL beaker using paperclips attached to the edges. Next, 4 g of bitumen was placed in each boat. The material was then placed in a forced air convection oven at 175 °C. Bitumen melted inside the boats but did not break through the treated fabric. No bitumen breakthrough was observed after 45 days. The bitumen was observed to slowly harden and solidify as volatile components evaporated, however. This test further attests to the long-term thermal stability and mechanical resilience of coated fabric substrates to retain bitumen without plastron collapse and leakage.

Bituminous deposits make up a significant portion of global oil reserves. The two largest heavy oil reserves are located in Canada and Venezuela in the Athabasca Oil Sands and the Orinoco Belt, respectively.^3,45^ The export of Canadian heavy oil to the U.S. Midwest and Gulf Coast for refining, and then export of asphalt from these refineries incurs substantial cost and is a major bottleneck to midstream transportation.^46,47^ Other heavy oil reserves are located in Iran, Iraq, Russia, and China, whereas^45–47^ the most notable heavy oil refineries that import their oil are located in The Netherlands, the U.A.E., Singapore, and South Korea.^46–48^ Based on these consideration, the most significant bottlenecks to heavy oil transportation are in overland transport from Canada to the U.S. Gulf Coast in pipelines and railcars, and then from the U.S. Gulf Coast in tankers. Similarly, midstream transportation from Middle Eastern heavy oil refineries (especially in the U.A.E.) and from European refineries in The Netherlands and Germany to customers by rail and sea remains a challenge.^45–48^ Application of liners within pipe walls, train cars, and barge containers have promise to greatly increase the efficiency and reduce costs of midstream transportation.^18^ The potential to apply the liners described herein to ocean-going vessels in particular would have substantial impact on the midstream transportation industry.^17^

## 4 Conclusions

Upon deposition of an electroless nickel composite coating embedding PTFE beads and subsequent surface functionalization with a PFOPA monolayer, a cotton fabric is rendered both superhydrophobic and superoleophobic to bitumen. The coated substrate demonstrates low roll-off angles and low contact angle hysteresis in dynamic contact angle measurements, which attest to minimal interfacial interactions with common fluids and enable facile gliding of both water and oil flow streams. The substrates are also observed to rapidly glide bitumen upon immersion and to resist oil fouling, which demonstrates their potential for applications requiring modification of the inner walls of pipelines and bitutainers. Electroless nickel deposition reinforces the cotton fabric and increases its strength but preserves sufficient flexibility such that the fabrics can be fashioned into free-standing receptacles or adjusted to follow the contours of vessels different forms. A coating thickness of ca. 2—4 μm of electroless nickel/PTFE is found to be optimal for fully utilizing the hierarchical texturation of the cotton substrate while retaining its flexibility and enabling reduction in surface energy upon surface functionalization with PFOPA. The coatings are thermally robust and resist degradation up to temperatures of 265 °C, well beyond the 220 °C operational temperature limit common to midstream transportation of bitumen. These results indicate a promising solution for the integration of treated fabric as liners in midstream transportation vessels, thereby mitigating the challenges of on-board coating, and bringing about substantial benefits in reducing product loss, minimizing the use of diluents and thermal jacketing, and greatly simplifying maintenance and cleaning operations.

## Supplementary Material





















## Notes

Additional SEM images, EDS maps, EDS spectra, statistical tests, XPS spectra, contact angle tables, viscosity profile, and bitumen wetting test images (PDF)
Video S1 showing dispensation of water droplet onto cotton fabric and attempt to retrieve (MP4)
Video S2 showing dipensation of water droplet onto PFOPA functionalized cotton fabric and attempt to retrieve (MP4)
Video S3 showing dispensation of water droplet onto EN-PTFE coated cotton fabric with PFOPA functionalization and attempt to retrieve (MP4)
Video S4 showing heavy oil removal after pouring on treated cotton fabric (MP4)
Video S5 showing heavy oil removal after immersion of treated cotton fabric (MP4)
Video S6 showing heavy oil removal after pouring on bare cotton fabric (MP4)
Video S7 showing heavy oil removal after immersion of bare cotton fabric (MP4)
Video S8 showing water removal after immersion of treated cotton fabric (MP4)
Video S9 showing water poured on treated cotton fabric (MP4)

## Abbreviations

APTMS: 3-aminopropyltrimethoxysilane
EDS: energy dispersive X-ray spectroscopy
EN: electroless nickel
FE-SEM: field-emission scanning electron microscopy
FTIR: Fourier transform infrared spectroscopy
PFOPA: 1*H*,1*H*,2*H*,2*H*-perfluorooctanephoshonic acid
PTFE: polytetrafluoroethylene
XPS: X-ray photoelectron spectroscopy
