# Supplementary material for: Retrofittable Flexible Fabric Liners with Surface-Functionalized Electroless Nickel Coatings for Midstream Transportation of Bitumen
Source: Energy Fuels. 2025 Jun 6;39(24):11625–35. doi: 10.1021/acs.energyfuels.5c01358 (PMC12186292; doi:10.1021/acs.energyfuels.5c01358)
Supplement: Supplementary file 1 [file ef5c01358_si_001.pdf]

Retrofittable Flexible Fabric Liners with Surface-Functionalized Electroless Nickel Coatings for Midstream Transportation of Bitumen

Joseph K. Cantrell,[1,2]‡ Lacey D. Douglas,[1,2]‡ Victor H. Balcorta,[1,2] Jason A. Weeden,[3] Shruti Hariyani,[1,2] Rachel H. Lee,[2] James A. Eaves,[1,2] Kaylyn Stewart,[1,2] Noah Pieniazek,[1,2] Matt Pharr,[2] Andrew A. Ezazi,[1,2,3]* Sarbajit Banerjee[1,2]*

[1]Department of Chemistry, Texas A&M University, College Station, Texas 77842-3012, United States

[2]Department of Materials Science and Engineering, Texas A&M University, College Station, Texas 77843-3003, United States

[3]Quiddity Products, 34011 Sunset Lane, Brookshire, Texas 77423-8517, United States

KEYWORDS: Bitumen, Heavy Crude, Midstream, Oleophobicity, Engineered Coatings, Viscous Oil, Wettability, Surface Modification

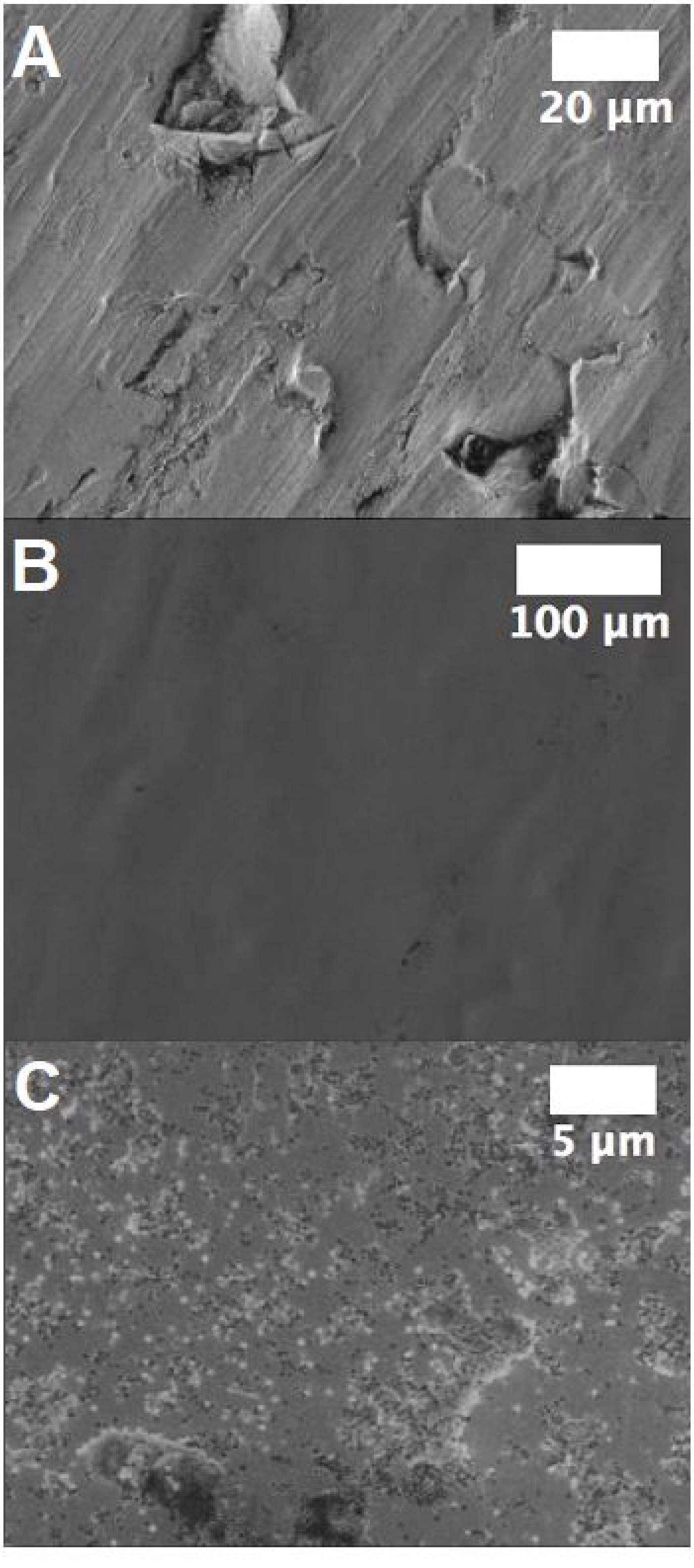

Figure S1. EN-PTFE Coating Flat Control. Scanning electron micrographs of (A) surface of A36 low-alloy steel; (B) the low alloy steel substrate after deposition of a ca. 12 µm thick electroless nickel/phosphorus composite coating; and (C) surface of the low alloy steel substrate after deposition of a ca. 12 µm electroless nickel/phosphorus composite coating embedding PTFE beads.

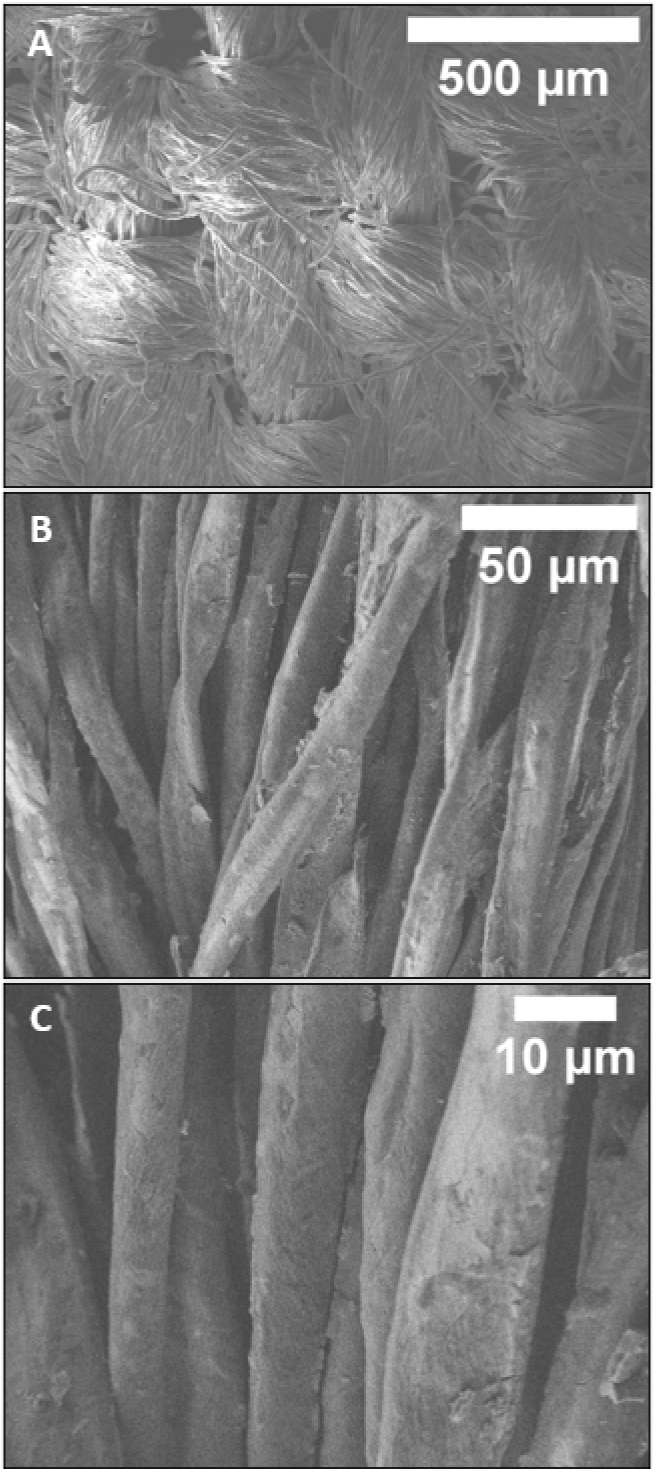

Figure S2. Cotton Fabric Control. Scanning electron micrographs of uncoated cotton substrate displaying intricate woven patterns.

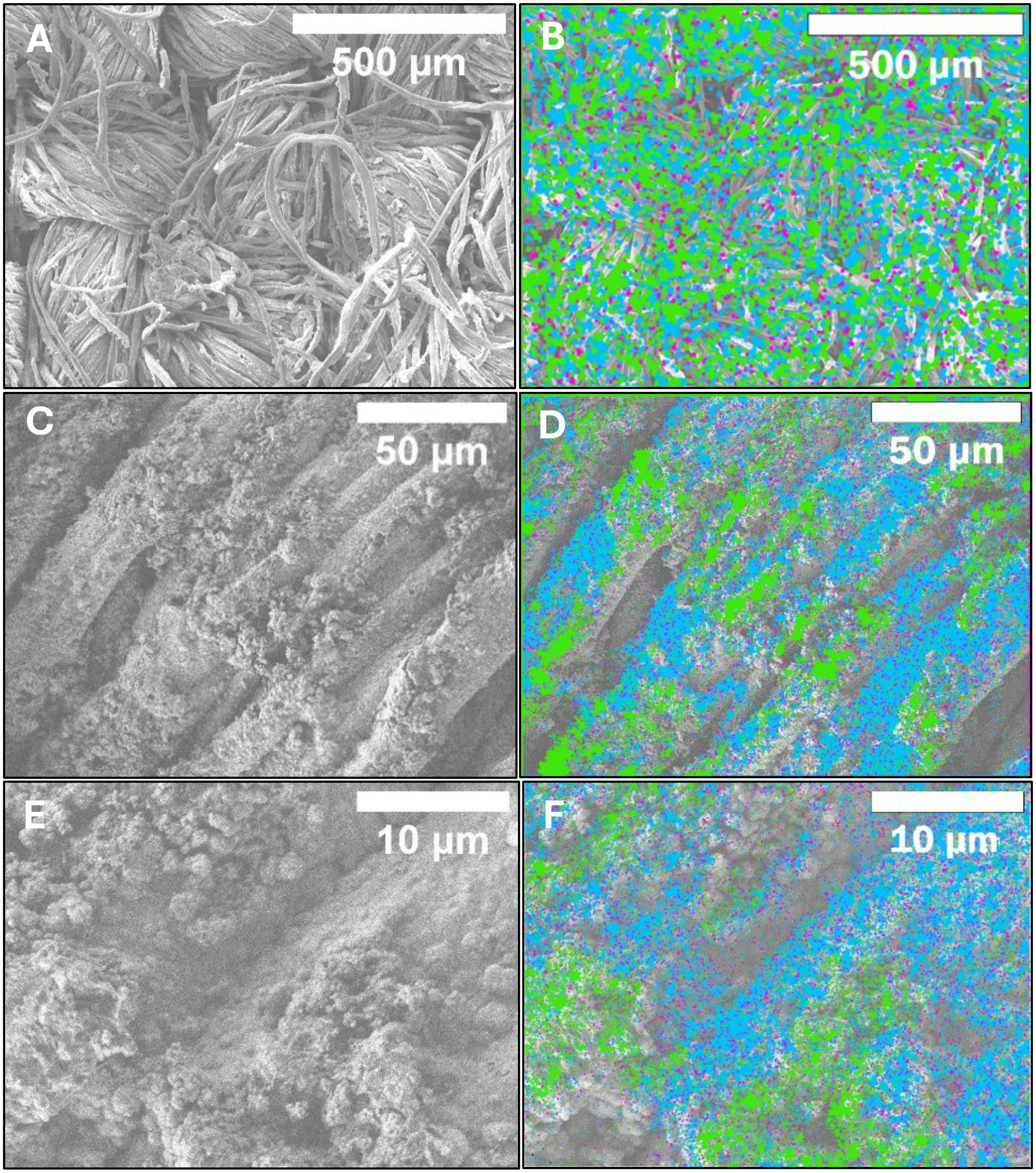

Figure S3. Cotton EN-PTFE Texturation Across Length Scales. Scanning electron micrographs and EDS maps of EN-PTFE coated cotton fabric samples with PFOPA functionalization. A, C, E) EN-PTFE texturization of fibers of a woven cotton fabric after 1 min of EN-PTFE coating at different magnifications. B, D, F) EDS maps corresponding to A, C, and E, respectively, where purple corresponds to phosphorus, blue corresponds to nickel, and green corresponds to fluorine. EDS spectra are shown in Figure S4.

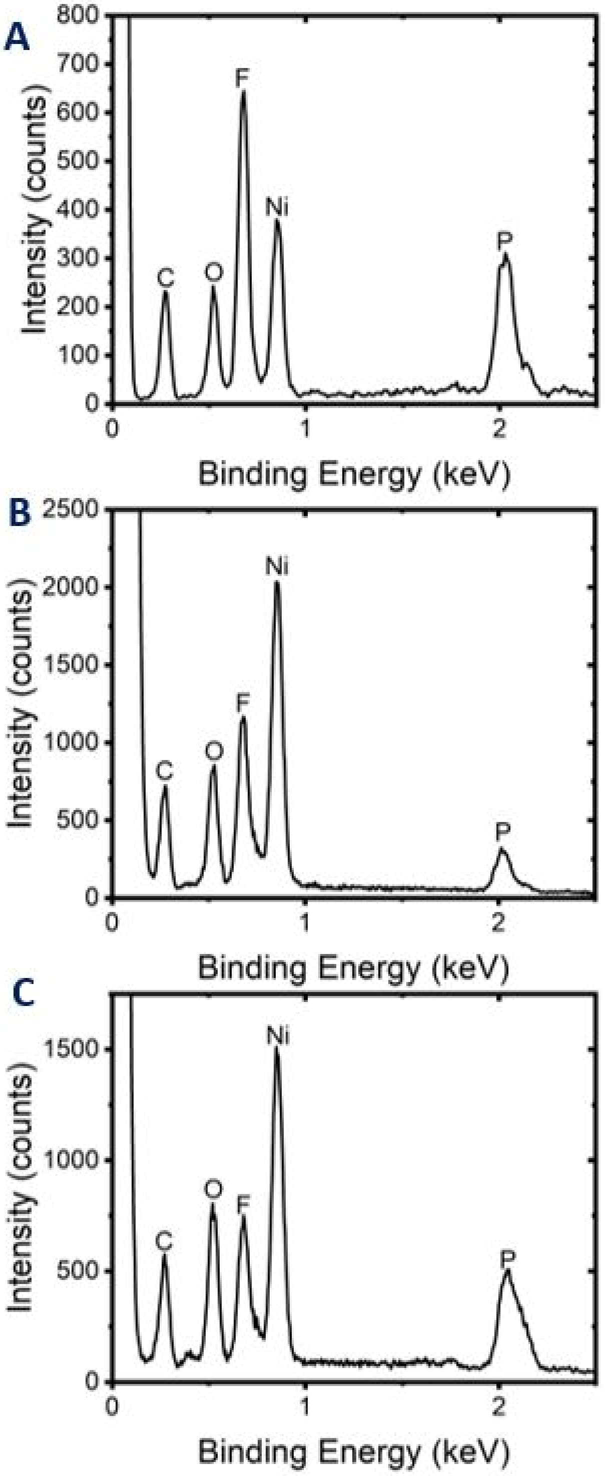

Figure S4. Labeled EDS plots corresponding to EDS maps shown in Figures S3 B, D, and F, respectively.

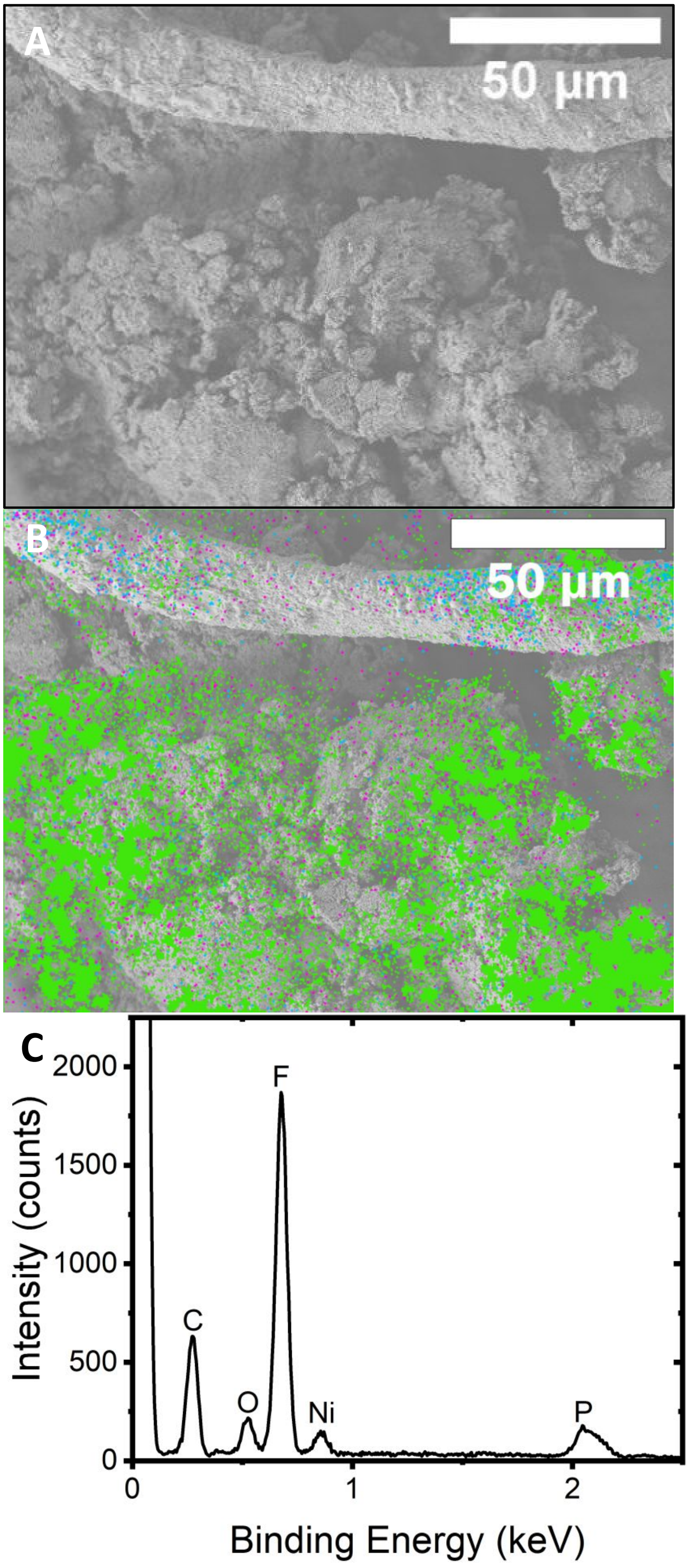

Figure S5. Scanning electron micrographs of EN-PTFE coated fabric functionalized with PFOPA with EDS mapping where purple corresponds to phosphorus, blue corresponds to nickel, and green corresponds to fluorine. A,B) dense coverage of PTFE beads discernible for thicker 10 min EN-PTFE coatings (with PFOPA functionalization), as corroborated by the EDS map. C) Labeled EDS spectrum.

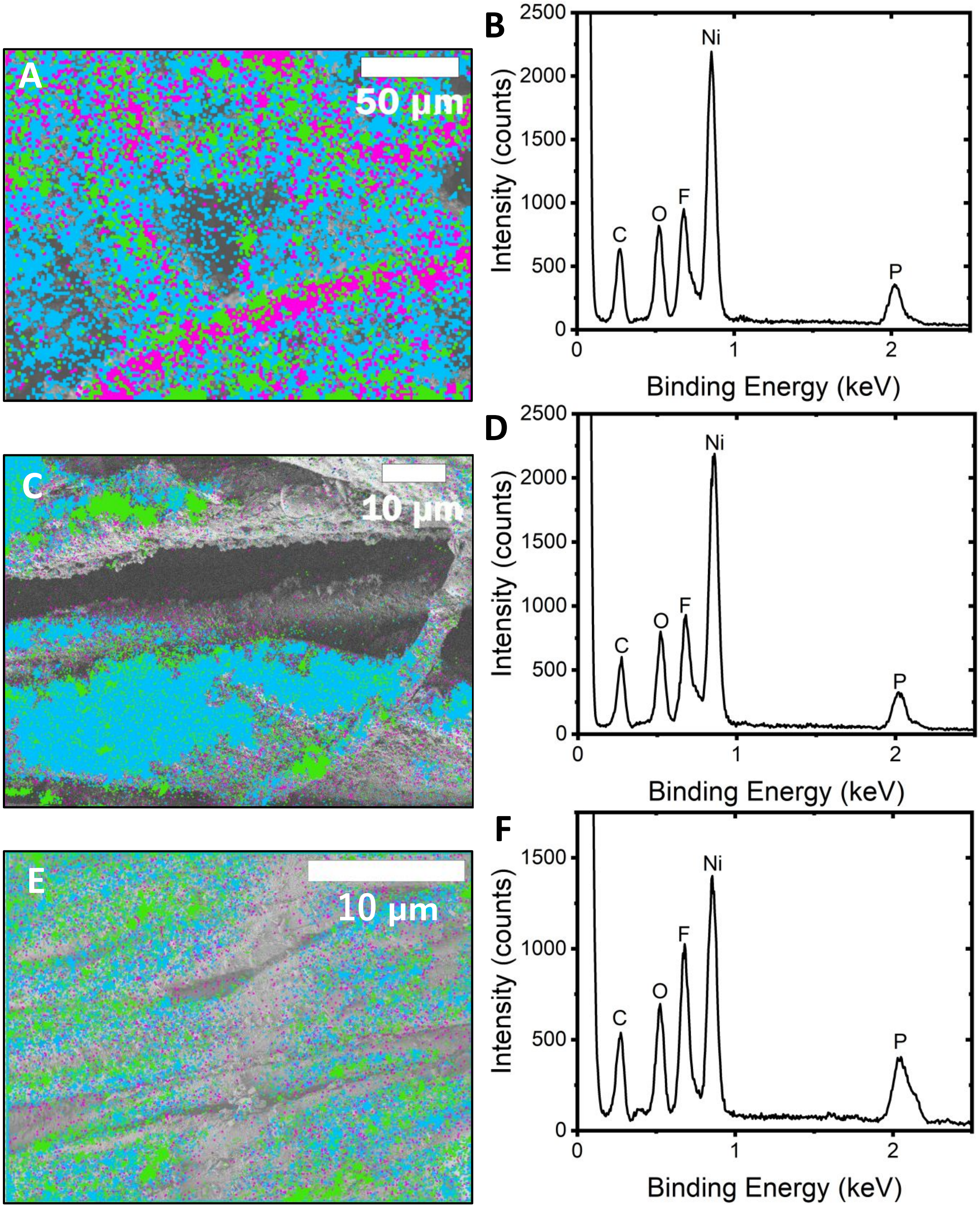

Figure S6. Scanning electron micrographs of EN-PTFE coated fabric functionalized with PFOPA with EDS mapping where purple corresponds to phosphorus, blue corresponds to nickel, and green corresponds to fluorine. A,C) EN-PTFE coating deposited on woven cotton for 1 min; E) EN-PTFE coating deposited on woven cotton for 45 min. **Figure 2** shows corresponding micrographs without EDS overlays. B, D, and F plot corresponding EDS spectra with labels.

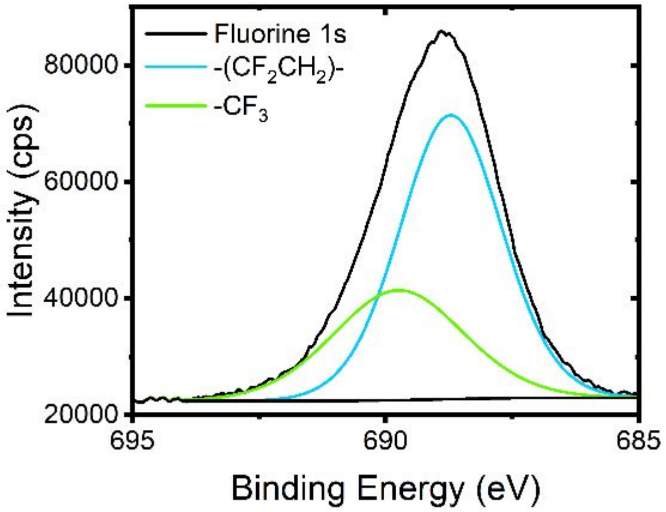

Figure S7. Core-level fluorine 1s XPS spectra of EN-PTFE-coated cotton fabric with PFOPA functionalization.

Table S1. P-values from t-tests comparing the breaking force and elongation at maximum force of PTFE Electroless Nickel-coated samples (deposited for 1, 10, and 60 min) to bare cotton fabric.

	P-value (Break Strain)	P-value (Break Force)
**1 min EN-PTFE**	0.015	0.002
**10 min EN-PTFE**	0.032	0.010
**60 min EN-PTFE**	0.008	0.012

Table S2. Characterization of Wettability. Water, heavy oil (synthetic bitumen, Puma Energy), and sweet light oil (Texas Permian Basin, specific gravity 0.8050) static contact angles measured for different thicknesses of EN-PTFE films without PFOPA surface functionalization. Dynamic water contact angles (advancing, receding, hysteresis) are also shown. The rightmost two columns list water roll-off angles of the substrates. Deposition times from 1—60 min noted in the first column correspond to EN thin film thicknesses from ca. 2 μm to 14 μm. For PFOPA functionalized samples see **Table 1**.

	Water Contact Angle (°)	Bitumen Contact Angle (°)	Light Sweet Oil Contact Angle (°)	Advancing Water Contact Angle (°)	Receding Water Contact Angle (°)	Water Contact Angle Hysteresis (°)	Water Dynamic Contact Angle (°)	Tilting Plate Roll-Off Angle (°)	Centimeter Drop Roll-Off Angle (°)
**1 min EN-PTFE**	151 ± 2	115 ± 2	0	151 ± 7	129 ± 16	22 ± 16	151 ± 2	35 ± 4	7 ± 2
**2 min EN-PTFE**	154 ± 4	122 ± 3	0	158 ± 15	131 ± 21	27 ± 21	154 ± 4	42 ± 7	12 ± 2
**5 min EN-PTFE**	153 ± 5	127 ± 3	0	152 ± 14	128 ± 6	24 ± 14	153 ± 5	47 ± 2	14 ± 3
**10 min EN-PTFE**	150 ± 3	133 ± 4	0	152 ± 10	131 ± 10	21 ± 10	150 ± 3	50 ± 8	13 ± 3
**20 min EN-PTFE**	154 ± 3	120 ± 2	0	154 ± 11	124 ± 19	30 ± 19	154 ± 3	>90	>90
**30 min EN-PTFE**	137 ± 2	135 ± 2	0	131 ± 29	106 ± 24	25 ± 29	137 ± 2	>90	>90
**45 min EN-PTFE**	123 ± 1	131 ± 5	0	133 ± 11	102 ± 10	31 ± 11	123 ± 1	>90	>90
**60 min EN-PTFE**	106 ± 2	123 ± 7	0	112 ± 14	81 ± 17	31 ± 17	106 ± 2	>90	>90

Table S3. Light sweet crude oil (Texas Permian Basin, specific gravity 0.8050) static contact angles, 20 μL water dynamic contact angles, and roll-off angles obtained by dropping a 20 μL droplet onto the substrate from a centimeter above. For water and heavy oil sessile contact angles, advancing and receding water contact angles, contact angle hysteresis, and tilting plate roll off angles, see **Table 1**.

	Light Sweet Oil Contact Angle (°)	Water Dynamic Contact Angle (°)	Centimeter Drop Roll-Off Angle (°)
**Bare Cotton**	0	24 ± 8	>90
**Cotton PFOPA**	79 ± 1	23 ± 12	>90
**1 min EN-PTFE PFOPA**	118 ± 3	11 ± 10	5 ± 3
**2 min EN-PTFE PFOPA**	124 ± 2	8 ± 7	7 ± 2
**5 min EN-PTFE PFOPA**	123 ± 5	9 ± 9	5 ± 1
**10 min EN-PTFE PFOPA**	120 ± 3	6 ± 5	8 ± 3
**20 min EN-PTFE PFOPA**	117 ± 6	9 ± 9	7 ± 2
**30 min EN-PTFE PFOPA**	113 ± 1	9 ± 9	4 ± 4
**45 min EN-PTFE PFOPA**	102 ± 3	7 ± 6	6 ± 3
**60 min EN-PTFE PFOPA**	97 ± 1	8 ± 8	9 ± 3

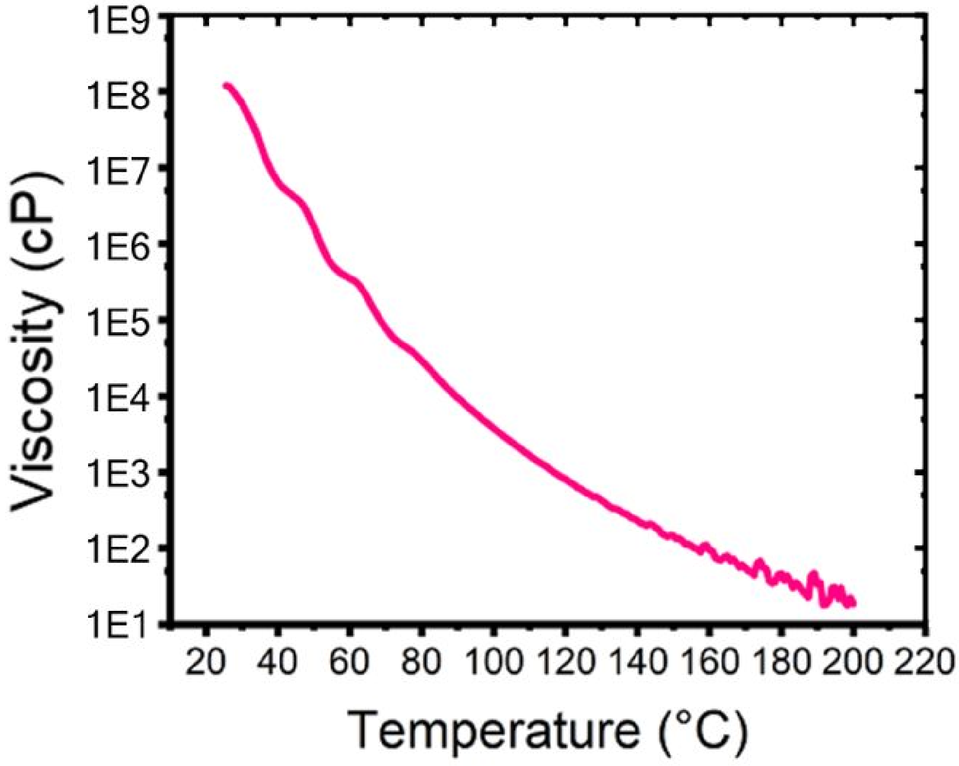

Figure S8. Viscosity profile showing viscosity as a function of temperature for Puma heavy crude oil used as a probe liquid.

Table S4. Water and heavy oil (.synthetic bitumen, Puma Energy) static contact angles measured for different thicknesses and kinds of functionalization of EN-PTFE films. Deposition times from 1—60 min noted in the first column correspond to EN thin film thicknesses from ca. 2 μm to 14 μm. Strong superhydrophobic character is retained for these surface treatments. Superoleophobic character is retained in the 27mM PFOA samples, but drops to oleophobic character in the 2.7mM PFOPA samples. For 27mM PFOPA functionalized samples see **Table 1**.

	**Water Contact Angle at Room Temperature (°)**		**Heavy Oil (Bitumen) Contact Angle at 175°C (°)**	
	**2.7mM PFOPA 24 h**	**27mM PFOA 24 h**	**2.7mM PFOPA 24 h**	**27mM PFOA 24 h**
**1 min EN-PTFE**	159 ± 2	150 ± 4	140 ± 4	148 ± 3
**2 min EN-PTFE**	154 ± 2	149 ± 3	140 ± 6	155 ± 4
**5 min EN-PTFE**	154 ± 1	149 ± 6	137 ± 6	152 ± 2
**10 min EN-PTFE**	152 ± 4	152 ± 3	139 ± 3	155 ± 5
**20 min EN-PTFE**	151 ± 1	147 ± 5	132 ± 6	149 ± 6
**30 min EN-PTFE**	152 ± 1	150 ± 4	137 ± 7	150 ± 6
**45 min EN-PTFE**	155 ± 0	148 ± 6	142 ± 5	142 ± 5
**60 min EN-PTFE**	153 ± 1	144 ± 3	137 ± 5	135 ± 9

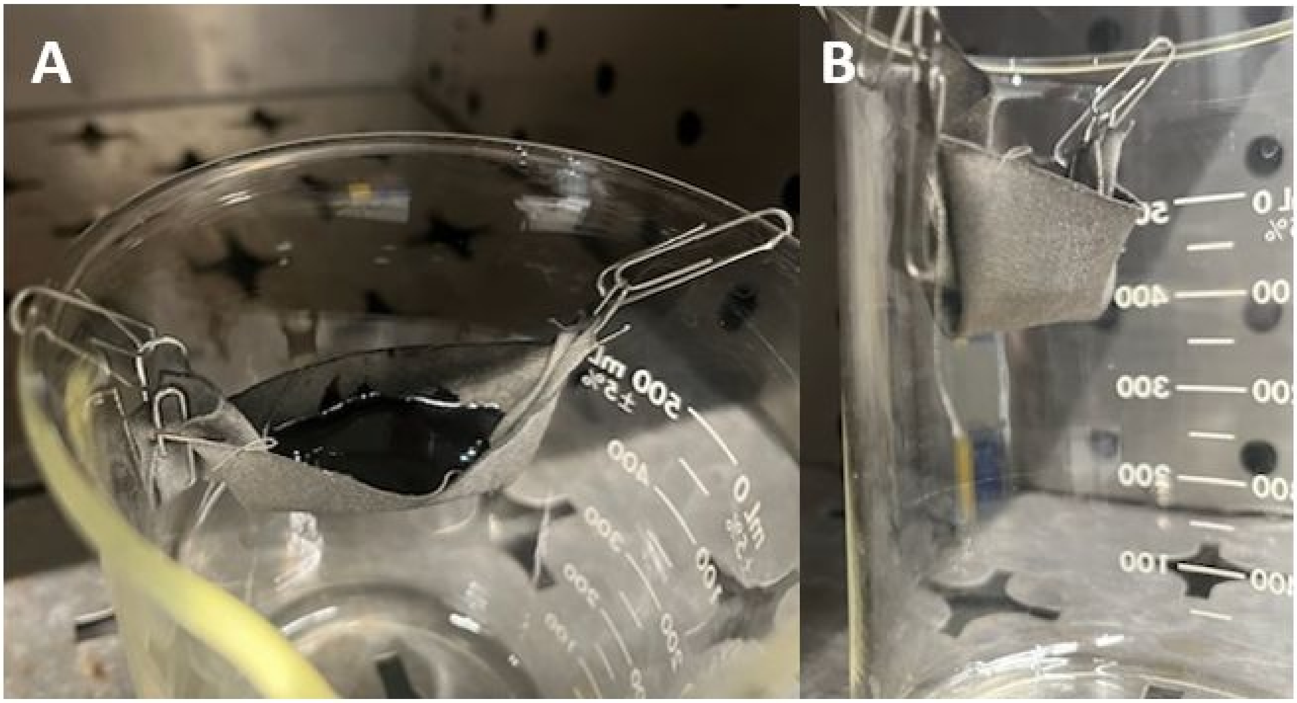

Figure S9. Experimental setup for 45-day bitumen breakthrough test. Cotton fabric with EN-PTFE coating and PFOPA functionalization is suspended in a boat shape filled with Puma bitumen and held at 175°. No bitumen was observed to permeate the fabric over the test period.

Video S1. Dispensation of a 20 μL water droplet onto a cotton fabric substrate and attempt to retrieve the droplet during advancing and receding contact angle experiments. The droplet initially resists wetting the fabric, but flash wets during measurement of receding contact angle.

Video S2. Dispensation of a 20 μL water droplet onto a PFOPA functionalized cotton fabric substrate and attempt to retrieve the droplet during advancing and receding contact angle experiments. The droplet resists flash wetting the fabric throughout the test, but adheres to the substrate and eventually will flash wet after several minutes.

Video S3. Dispensation of a 20 μL water droplet onto an EN-PTFE coated cotton fabric substrate functionalized with PFOPA and attempt to retrieve the droplet during advancing and receding contact angle experiments. The droplet resists wetting the fabric, maintaining high contact angles during advancing and receding measurements and separates from the fabric as the test concludes.

Video S4. Heavy oil (at 250°C) removal after pouring on treated cotton fabric (10 min PTFE electroless nickel with 27 mM PFOPA functionalization). All oil quickly slides off except for some remnant droplets that are pinned but do not wet the surface.

Video S5. Heavy oil (at 250°C) removal after immersion of treated cotton fabric (10 min PTFE electroless nickel with 27 mM PFOPA functionalization). Heavy oil cannot wet the surface and leaves no residue except as droplets on the bottom edge.

Video S6. Heavy oil (at 250°C) removal after pouring on cotton fabric. The oil wets and adheres strongly to the surface.

Video S7. Heavy oil (at 250°C) removal after immersion of bare cotton fabric. The oil wets and adheres strongly to the surface.

Video S8. Water removal after immersion of treated cotton fabric (10 min PTFE electroless nickel with 27 mM PFOPA functionalization) at room temperature. Formation of the plastronic air layer can be observed and the surface remains dry after immersion.

Video S9. Water poured on treated cotton fabric (10 min PTFE electroless nickel with 27 mM PFOPA funcrionalization) at room temperature. The water immediately balls up and rolls off the surface.
